# Pectin and Xylan Biosynthesis in Poplar: Implications and Opportunities for Biofuels Production

**DOI:** 10.3389/fpls.2021.712083

**Published:** 2021-08-19

**Authors:** Joshua A. Schultz, Heather D. Coleman

**Affiliations:** Department of Biology, Syracuse University, Syracuse, NY, United States

**Keywords:** GT8D, secondary cell wall, irx8, reducing end sequence, hemicellulose

## Abstract

A potential method by which society's reliance on fossil fuels can be lessened is *via* the large-scale utilization of biofuels derived from the secondary cell walls of woody plants; however, there remain a number of technical challenges to the large-scale production of biofuels. Many of these challenges emerge from the underlying complexity of the secondary cell wall. The challenges associated with lignin have been well explored elsewhere, but the dicot cell wall components of hemicellulose and pectin also present a number of difficulties. Here, we provide an overview of the research wherein pectin and xylan biosynthesis has been altered, along with investigations on the function of *irregular xylem 8* (*IRX8)* and *glycosyltransferase 8D* (*GT8D*), genes putatively involved in xylan and pectin synthesis. Additionally, we provide an analysis of the evidence in support of two hypotheses regarding *GT8D* and conclude that while there is evidence to lend credence to these hypotheses, there are still questions that require further research and examination.

## Introduction

Reliance on fossil fuels is unsustainable due to climate change (IPCC, 2018). Transitioning to environmentally friendly energy sources has become an urgent necessity. One alternative energy source with the potential to mitigate damage caused by climate change is biofuel. Support for the transition away from fossil fuels and toward biofuels is bolstered by the potential stimulation of rural economies (Kleinschmidt, 2007; Somerville, 2007). Nevertheless, a multiplicity of considerations must be taken into account in order to produce biofuels in an economic and efficient manner. Currently, corn is the main source of bioethanol in the United States, but only reduces greenhouse gas (GHG) emissions by 13% (Farrell et al., 2006). In contrast, cellulosic ethanol derived from plants such as hybrid poplar can reduce net GHG emissions to nearly zero (Solomon et al., 2007). Researchers found that in a poplar short-rotation coppice in Belgium, the bioenergy plantation's high CO_2_ uptake outweighed non-CO_2_ greenhouse gas emissions (Horemans et al., 2019). The chemical properties and ubiquity of the secondary cell wall (SCW) have led to the SCW being marked as a biofuel source of interest. However, a number of challenges facing second-generation biofuels emerge from the underlying intricacy of the SCW and the individual constituent biopolymers of which it is composed. A comprehensive understanding of SCW biosynthesis is critical to optimize the chemical composition of the SCW for biofuel production.

## Secondary Cell Wall

Key to understanding the SCW is the structure and function of its components. The SCW is mainly composed of cellulose, hemicellulose (in poplar, predominantly xylan) and lignin. Although not considered a major SCW component, recent work has shown the importance of pectin in relation to biofuels (Biswal et al., 2018a; Yang et al., 2020).

### Cellulose

Cellulose consists of β-(1,4)-linked glucose monomers and contributes significantly to plant structural integrity (Klemm et al., 2005). It is synthesized at the plasma membrane where a hexameric rosette of cellulose synthase proteins (encoded by *CesA* genes) form the cellulose synthase complex which catalyzes the biogenesis of multiple cellulose polymers (Kimura et al., 1999).

### Lignin

Lignin is a complex biopolymer made from cross-linked molecules derived from three precursor monolignols (Vanholme et al., 2010; Kumar et al., 2016) which are oxidized by peroxidases and/or laccases into the lignin polymer as *p*-hydroxyphenyl, guaiacyl, and syringyl *via* radical coupling in the SCW (Bose et al., 2009). Lignin plays a crucial role in forming the cell walls of woody plants and has function in reinforcing cell wall rigidity and facilitating liquid transportation (Hofrichter, 2002; Zeng et al., 2014). Additionally, lignin contributes to cell wall recalcitrance; one study found that poplar with reduced lignin content could potentially decrease overall bioethanol costs by ~41% (Littlewood et al., 2014).

### Hemicellulose

Hemicelluloses are composed of β-(1,4)-linked sugar backbones with an equatorial 3D conformation (Scheller and Ulvskov, 2010). Xylan in particular is comprised of a backbone of xylose monomers with a profusion of chemical substituents such as acetyl, glucuronic acid (GlcA), 4-*O*-methylglucuronic acid, and arabinose side groups (Rennie and Scheller, 2014). Attached to the end of the xylan backbone is the reducing end sequence (RES), an oligosaccharide composed of xylose, rhamnose, and galacturonic acid (Scheller and Ulvskov, 2010). Glucuronoxylan is the predominant type of hemicellulose in angiosperms; it is composed of a xylose chain substituted with glucuronic acid (often 4-*O*-methylated) and acetyl groups.

Xylan biosynthesis occurs within the medial Golgi. Glycosyltransferases, enzymes that use nucleotide sugars as a substrate to catalyze the formation of glycosidic linkages between sugars, are the main proteins involved in synthesizing xylan (Keegstra and Raikhel, 2001). Genes from the GT43 gene family, IRX9/IRX9L (Lee et al., 2010; Wu et al., 2010) and IRX14/IRX14L (Keppler and Showalter, 2010), and genes from the GT47 gene family, IRX10/IRX10L (Brown et al., 2009; Wu et al., 2009), have been implicated in the elongation of the xylan backbone. After its manufacture in the Golgi apparatus, xylan is trafficked to the plasma membrane and incorporated into the growing SCW *via* exocytosis (Rennie and Scheller, 2014). Once assimilated into the SCW, xylan fulfills various structural and functional roles at the molecular, organismal, and ecological level. At the molecular level, xylan is involved in cross-linking cellulose microfibrils; consequently, this has the effect of stabilizing the cellulose microfibrils and ensuring that they are properly oriented (Wierzbicki et al., 2019). At the organismal level, xylan facilitates normal plant development, and at the ecological level, it increases cell wall recalcitrance, thereby protecting plants against biotic stress-inducing agents such as pathogens and herbivores (Rennie and Scheller, 2014). Recent work has also further elucidated the acetylation and methylation of xylan (Urbanowicz et al., 2014; Grantham et al., 2017; Pawar et al., 2017; Yang et al., 2017; Zhong et al., 2018; Lunin et al., 2020).

### Pectin

Pectins, structurally and functionally complex polysaccharides principally made of galacturonic acid, are synthesized in the Golgi apparatus and transported to the growing cell wall *via* exocytosis (Mohnen, 2008). There are multiple types of pectins including: homogalacturonan (HG) pectins, rhamnogalacturonan I (RG-I) pectins, rhamnogalacturonan II (RG-II) pectins, and xylogalacturonan (XGA) pectins. Homogalacturonan consists of a linear chain of α-(1-4)-linked D-galacturonic acid, partially methylesterified at the C-6 carboxyl and/or acetylated at O2 or O3 (Mohnen, 2008). Rhamnogalacturonan I contains a chain of alternating galacturonic acid and rhamnose, with various side chains mainly galactose, arabinose, and xylose (O'Neill et al., 2004). Rhamnogalacturonan II is a highly branched polysaccharide that contains the same backbone as homogalacturonan (O'Neill et al., 2004). Xylogalacturonan is at its core HG, but with additional β-1,3-xylosyl side groups (Mohnen, 2008). Pectin plays roles in cell signaling, defense and maintenance of cell wall structure, and is also a key player in cell-cell adhesion (Mohnen, 2008). In this role, HG is de-methylesterified, causing it to become negatively charged. The negatively charged homogalacturonan molecules bond with Ca^2+^ ions, forming HG:HG salt bridges (Jarvis et al., 2003). In addition, the apiose groups of RG-II polysaccharides can form borate diesters, resulting in dimerization (Kobayashi et al., 1996; O'Neill et al., 2004; Yang et al., 2020). Because of its chemical properties, pectin contributes to the recalcitrance of plant biomass to the conversion of biofuels. RG-II borate diester cross-linking and HG:HG salt bridges formed by pectins results in increased cell wall recalcitrance (Biswal et al., 2018a). Consequently, downregulating pectin increases the accessibility of cell wall biomass to enzymatic degradation (Biswal et al., 2018a).

## Biofuels and Cell Wall Recalcitrance

Xylan contributes to difficulties associated with second-generation biofuel production, in part because of the five-carbon (C_5_) sugar monomers. Fermentation of xylose is an arduous process since yeast lack the natural ability to ferment C_5_ sugars (Rennie and Scheller, 2014) and C_5_ sugars inhibit the reaction that ferments six-carbon (C_6_) sugars (Brandon et al., 2020).

An additional challenge is the release of the acetyl groups bonded to xylan, which produces acetic acid, altering the pH of the fermentation media (Rennie and Scheller, 2014; Rastogi and Shrivastava, 2017; Yusuf and Gaur, 2017). This has an inhibitory effect on ethanologenic biocatalysts as acetic acid molecules travel across the plasma membrane and act as a protonophore, acidifying the cytoplasm (McMillan, 1994; Lawford and Rousseau, 1998). Coupled with this, acetyl groups sterically hinder hydrolytic enzymes responsible for breaking down glycosidic bonds in complex carbohydrates (Biely, 2012). As a result, various pre-treatments as well as enzymatic, microbial, and chemical treatments must be used during saccharification and fermentation processes, reducing the cost-effectiveness and efficiency of biofuel production (Wierzbicki et al., 2019). Elucidating the genetic and cellular mechanisms that modulate xylan biosynthesis is essential for the development of biotechnological solutions to recalcitrance due to xylan.

A variety of approaches have been used to modify hemicelluloses to rectify these challenges. Loss-of-function irregular xylem (*irx*) mutations in xylan biosynthetic genes come with the vicissitude of causing the xylem vessels in the plants to collapse, thereby stunting growth (Brown et al., 2005). Despite these setbacks, researchers working with *Arabidopsis thaliana* have refined the means by which plants with reduced xylan content are produced. Using mutants in genes encoding glycosyltransferases (e.g., *IRX7* and *IRX8*), they reinstated xylan expression in the vessel tissue by utilizing vessel-specific transcription factor promoters (*VND6* or *VND7)*, thereby reducing total xylan content but retaining normal levels in the vessels (Petersen et al., 2012).

*Populus* species have two galacturonosyltransferase 12 (GAUT12) orthologs: *GAUT12.1* (GT8D) and *GAUT12.2*. Unlike *irx8 Arabidopsis* mutants, *Populus deltoides GAUT12.1* knockdown (*GAUT12.1*-KD) lines were phenotypically normal, and in fact most lines increased in height relative to controls (Biswal et al., 2015). The downregulation of *PdGAUT12.1* led to a reduction in galacturonic acid and xylose (Biswal et al., 2015). *GAUT4* (a HG biosynthesis gene) downregulation *via* RNAi resulted in reduced HG, RG-II, cell wall calcium, and cell wall boron along with a seven-fold increase in saccharification and ethanol production and a six-fold increase in biomass yield compared to control plants (Biswal et al., 2018a). Work in other species has also indicated improvements in quality of biomass for biofuels, including assessment of mutants in Arabidopsis where a decrease in de-methylesterified HG was correlated with improved degradability of cellulose (Francocci et al., 2013) and switchgrass with *GAUT4-*KD lines where reduced HG and RG content and cross-linking resulted in improved sugar yield (Holwerda et al., 2019; Li et al., 2019). These results demonstrate that decreasing pectin and xylan levels can improve cell wall properties without compromising plant structural integrity.

Alternative approaches have focused on altering sidechains on the xylan backbone to reduce biomass recalcitrance. A reduction in the quantity of acetyl groups bound to xylan is highly preferable, however many mutants with reduced xylan acetylation manifest phenotypic characteristics (e.g., stunted growth) that render them sub-optimal for biotechnological applications (Manabe et al., 2013; Yuan et al., 2013). However, new techniques have been developed to reduce xylan acetylation without stunting growth. *A. thaliana* plants with mutations in the TBL29 gene have 60% less xylan acetylation, but biomass yields are significantly reduced (Xiong et al., 2015). To compensate for the reduced acetylation, Xiong et al. (2015) successfully restored normal growth by overexpressing *GUX1* in *tbl29* mutants to increase the number of glucuronic acid (GlcA) substitutions. Mutation of *GXMT1*, which encodes a GX-specific 4-O-methyltransferase responsible for methylating GlcA residues in GX, resulted in decreased methylation of glucuronic acid and improved release of xylose following mild hydrothermal pre-treatment (Urbanowicz et al., 2012).

An important feature of xylan is the reducing end sequence (RES), which is hypothesized to act as a primer or a terminator (Lee et al., 2007; York and O'Neill, 2008). There are a number of genes hypothesized to be involved in RES synthesis. Of particular interest here is *GT8D*, the poplar ortholog of *IRX8*/*GAUT12*, and part of the GT8 family. These enzymes catalyze glycosidic linkages, and *GT8D* is most likely involved in the formation of the tetrasaccharide RES in xylan (Lee et al., 2011). The RES is comprised of β-Xyl-(1,3)-α-Rha-(1,2)-α-GalA-(1,4)-Xyl. IRX8 is hypothesized to catalyze the addition of galacturonic acid to the RES (Peña et al., 2007). Other genes implicated in RES synthesis include *IRX7, IRX7L*, and *PARVUS* (Brown et al., 2007; Petersen et al., 2012). A potential approach to modification of hemicellulose for improved biofuels production involves the RES.

### Functions of GT8D

*GT8D*, discussed above, is hypothesized to encode an enzyme that catalyzes the addition of galacturonic acid to pectin molecules and to the RES of xylan (Dual Function Hypothesis). *GT8D* is part of the *GAUT* gene family (Biswal et al., 2015; Kumar et al., 2019) and has 61% amino acid sequence similarity with GAUT1 (Biswal et al., 2015). GAUT1 has been demonstrated to add galacturonic acid to pectin (Sterling et al., 2006). Thus, it is possible that GT8D is also able to catalyze the addition of galacturonic acid to pectin. Work on the function of GT8D also lends credence to the claim that GT8D is responsible for the addition of galacturonic acid to pectin and the xylan RES. Downregulating *P. deltoides GAUT12.1* (*GT8D*) led to a reduction in galacturonic acid and xylose (Biswal et al., 2015). In *A. thaliana, irx8* mutants had significantly reduced xylan and homogalacturonan levels (Persson et al., 2007). These results implicate GT8D in both pectin and xylan biosynthesis. The silencing of GT8D resulting in a reduction in galacturonic acid levels is consistent with the claim that it adds galacturonic acid to pectin, and the lowered xylose levels are consistent with the claim that GT8D is involved in synthesizing the RES because if the RES cannot be completed, then the cells would be deficient in xylan. Additionally, overexpression of GAUT12.1 in *P. deltoides* yielded a simultaneous increase in xylan and homogalacturonan (Biswal et al., 2018b). Although this result is consistent with the claim that GAUT12.1 synthesizes part of the RES, the authors suggest GAUT12.1 is involved in the formation of a pectic glycan necessary for xylan biosynthesis (Biswal et al., 2018b). They reasoned that if only xylose levels were increased in the alcohol insoluble residue from the transgenics that overexpressed GAUT12.1, then that would support the hypothesis that GAUT12.1 is involved in synthesizing the RES, but if both xylose and galacturonic acid levels increased, then that would support the hypothesis that GAUT12.1 synthesizes a pectic glycan necessary for xylan biosynthesis (Biswal et al., 2018b). However, the increase in xylose and galacturonic acid is an experimental result that is also consistent with the predictions of the Dual Function Hypothesis.

### Formation and Function of the Reducing End Sequence

As discussed, the RES is hypothesized to function as a primer that initiates the elongation of the xylan chain (Primer Hypothesis). This hypothesis is founded on the following: firstly, *in vitro* biochemical analysis of xylan found that the addition of xylose subunits by IRX10-L transpires from the reducing end toward the non-reducing end, thus rendering the claim that the RES acts as a terminator unlikely (Urbanowicz et al., 2014; Smith et al., 2017). Secondly, whether the RES is a primer or a terminator, both contingencies entail certain predictions. If the RES is a terminator, then *irx8* loss-of-function mutants would be unable to fully halt xylan synthesis. If this is the case, the plant would most likely die if its cells synthesized xylan polysaccharides indefinitely. Alternatively, the plant cells might be able to halt the synthesis of xylan without the complete RES. In either case, one would not expect to see reduced xylan levels; the plant would either die or maintain normal xylan levels. Moreover, if the RES is a primer, one would expect to see *irx8* mutants that are deficient in xylan, because the cells would be unable to initiate xylan synthesis without the RES or would require an additional enzyme to take over this capacity (possible with duplication of genes). It follows from these premises that *irx8* mutants would either have normal xylan levels, die, or be deficient in xylan. As *irx8* mutants are deficient in xylan (Persson et al., 2007), it follows that the RES is unlikely to be a terminator. It is worth noting that despite the evidence in support of the Primer Hypothesis, there is some support for the hypothesis that the RES is a terminator. York and O'Neill (2008) argue that the RES is more likely to act as a terminator because action as a primer is seemingly inconsistent with its role in regulating xylan chain length. Additionally, Peña et al. (2007) speculate that chain termination could still occur without RES synthesis at lower frequencies *via* the transfer of nascent xylan chains to water.

Further support for the Primer Hypothesis includes the parallels between xylan biosynthesis and animal glycosaminoglycan (GAG) biosynthesis. Enzyme families involved in xylan biosynthesis (GT47 and GT43) are also involved in the biosynthesis of GAGs such as heparan sulfate and chondroitin sulfate (Smith et al., 2017). In addition, Lee et al. (2007), who propose a model in which the RES acts as a primer, argue that the fact that PARVUS, a protein necessary for RES synthesis, is localized in the endoplasmic reticulum (ER) rather than the Golgi apparatus provides evidence favoring the RES acting as a primer for xylan biosynthesis. They reason that since PARVUS is ER-localized, it probably catalyzes an enzymatic step prior to the step catalyzed by IRX8, which is Golgi-localized. Lee et al. (2007) hypothesize that PARVUS initiates the creation of the RES by catalyzing the addition of the reducing xylose residue to an unknown acceptor in the ER, with the subsequent enzymatic steps taking place in the Golgi body, and further compare this mechanism of xylan synthesis to that of GAG synthesis. They note that not only do GAGs need a tetrasaccharide primer, but also that the synthesis of the primer starts in the ER *via* the addition of the reducing xylose residue to a protein, with the ensuing primer synthesis steps occurring in the Golgi body (Prydz and Dalen, 2000; Lee et al., 2007; Yu and Linhardt, 2018). Additionally, the RES in heparan sulfate attaches to a protein necessary for transport to the cell wall (Kreuger and Kjellén, 2012). Wierzbicki et al. (2019) hypothesized that xylan may also attach to a protein required for transport. They note that some evidence for this already exists since arabinogalactan proteins (AGPs) called ARABINOXYLAN PECTIN ARABINOGALACTAN PROTEIN1 (APAP1) were found to have been linked to PCW xylans (Tan et al., 2013). Wierzbicki et al. (2019) suggested that if xylan needs to be attached to an AGP in order to be transported to the cell wall, a lack of transport to the cell wall could explain why plants with mutations in RES biosynthetic genes have SCWs that are deficient in xylan.

## Conclusion

A better understanding of the function of GT8D and the RES has the potential to facilitate the development of biotechnological tools that can reduce recalcitrance due to xylan. The evidence in the available scientific literature lends credence to the Dual Function Hypothesis and supports the Primer Hypothesis, but some questions require further examination regarding the RES. More research must be conducted to fully elucidate its function. A clear understanding of both the formation and function of the RES could lead to the development of mechanisms to modulate xylan and pectin content.

## Author Contributions

JS and HC conceptualized the manuscript. JS wrote the draft manuscript. HC reviewed and edited the manuscript. Both authors contributed to the article and approved the submitted version.

## Conflict of Interest

The authors declare that the research was conducted in the absence of any commercial or financial relationships that could be construed as a potential conflict of interest.

## Publisher's Note

All claims expressed in this article are solely those of the authors and do not necessarily represent those of their affiliated organizations, or those of the publisher, the editors and the reviewers. Any product that may be evaluated in this article, or claim that may be made by its manufacturer, is not guaranteed or endorsed by the publisher.
